# Polymer-Modified Liposomes for Drug Delivery: From Fundamentals to Applications

**DOI:** 10.3390/pharmaceutics14040778

**Published:** 2022-04-02

**Authors:** Yifeng Cao, Xinyan Dong, Xuepeng Chen

**Affiliations:** 1 Department of Electronic Chemicals, Institute of Zhejiang University-Quzhou, Quzhou 324000, China; 2 School of Biological and Chemical Engineering, NingboTech University, Ningbo 315100, China; dxyan@zju.edu.cn; 3 The Affiliated Hospital of Stomatology, School of Stomatology, Zhejiang University School of Medicine, Clinical Research Center for Oral Diseases of Zhejiang Province, Key Laboratory of Oral Biomedical Research of Zhejiang Province, Cancer Center of Zhejiang University, Hangzhou 310006, China

**Keywords:** liposome, polymer, drug delivery, long circulation, polymer–lipid conjugates, targeting, stimulus-responsive

## Abstract

Liposomes are highly advantageous platforms for drug delivery. To improve the colloidal stability and avoid rapid uptake by the mononuclear phagocytic system of conventional liposomes while controlling the release of encapsulated agents, modification of liposomes with well-designed polymers to modulate the physiological, particularly the interfacial properties of the drug carriers, has been intensively investigated. Briefly, polymers are incorporated into liposomes mainly using “grafting” or “coating”, defined according to the configuration of polymers at the surface. Polymer-modified liposomes preserve the advantages of liposomes as drug-delivery carriers and possess specific functionality from the polymers, such as long circulation, precise targeting, and stimulus-responsiveness, thereby resulting in improved pharmacokinetics, biodistribution, toxicity, and therapeutic efficacy. In this review, we summarize the progress in polymer-modified liposomes for drug delivery, focusing on the change in physiological properties of liposomes and factors influencing the overall therapeutic efficacy.

## 1. Introduction

**Figure 1:**
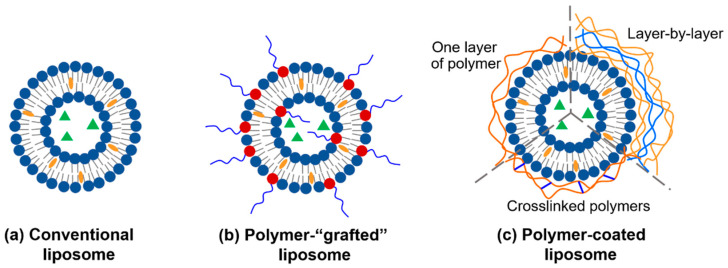
Liposomes for drug delivery: (**a**) conventional liposome, (**b**) polymer-“grafted” liposome, and (**c**) polymer-coated liposome, where liposomes are coated by a layer of adsorbed polymer, by the layer-by-layer (LbL) assembly, or by crosslinked polymers (polymer-“caged”). Liposomes can encapsulate hydrophobic drugs (orange ovals) in the hydrophobic region and hydrophilic ones (green triangles) in the interior aqueous region.

Efficient drug delivery systems are highly demanded in treating human diseases by satisfying pharmacological properties and therapeutic efficiencies [1]. Liposomes (Figure 1), first discovered in 1961 and reported in 1965 by Bangham et al. [2], are vesicles of closed lipid bilayer(s) composed mainly of phospholipids (PLs) with or without cholesterol (Chol), the major components of mammalian cell membranes. The hydrophobic interior of the lipid bilayer and the internal aqueous phase of liposomes enable the encapsulation of respective hydrophobic and hydrophilic drugs with relatively high drug encapsulation. Together with their good biocompatibility, tunable physicochemical and biophysical properties, as well as controlled release of drugs, liposomes are among the most feasible and extensively studied drug-delivery systems that are promising for advanced drug delivery in the treatment of cancers and other diseases [3,4]. More significantly, a number of liposomal formulations have been approved for clinical practice by the Food and Drug Administration (FDA) of the United States and the European Medicines Agency (EMA) of Europe since the first nanosized drug delivery system based on poly(ethylene glycol)-modified (PEGylated) liposome, Doxil^®^, was approved in 1995 [5,6]. More promising formulations are under clinical trials [7]. Meanwhile, more administration routes, such as nasal [8], inner ear [9], skin [10], oral [11], intra-articular [4,12], and gastrointestinal drug delivery [13], have been designed. Combination therapy by co-delivery of multiple therapeutic agents with liposomal formulations has also become an emerging research area [14].

## 2. Conventional Liposomes as Drug-Delivery Carriers

**Figure 2:**
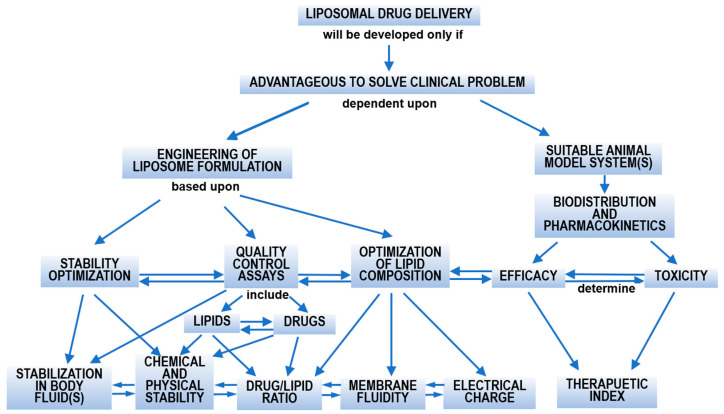
Concept map on developing liposomal drug delivery systems. Adapted from Ref. [15], published by Elsevier, 1993.

The concept in designing liposomal formulations (Figure 2) [15] clearly depicts the complexity. The vesicle composition plays a central role in determining the physicochemical properties, surface structure, interactive properties, and in vivo performance, including pharmacokinetics, biodistribution, toxicity, and therapeutic efficacy.

**Figure 3:**
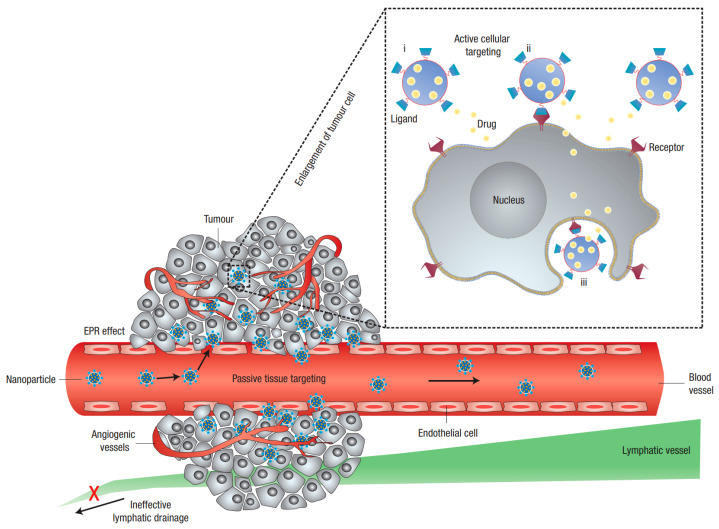
Nanoparticles, including liposomes, passively penetrate the blood vessel through the gaps between endothelial cells by the enhanced permeation and retention (EPR) effect at the tumor site, followed by endocytosis by the tumor cell. Adapted with permission from Ref. [22]. Published by Nature Publishing Group, 2007.

The physicochemical properties of liposomes, including phase state of the membranes, surface charge, and rigidity of lipid membranes, are determined by the lipid composition and size distribution of the vesicles and impact the physiological and pharmacokinetics performances of liposomal formulations, and consequently the efficacy of encapsulated drugs and toxicity [3,16]. The bilayer composition of conventional liposomes (Figure 1a)—also called the first-generation liposomes—is purely lipid-based. The major component of conventional liposomes is zwitterionic PL, including phosphatidylcholine (PC), phosphatidylethanolamine (PE), and sphingomyelin (SM), as well as nonionic lipids, such as cholesterol. The phase state of the bilayer, which characterizes the permeability, is determined by the structure of PLs (including headgroup, alkyl chain length, degree of unsaturation, and the ratio of cholesterol inserted in the bilayer [17]). A small amount of charged PLs, including negatively charged phosphatidylserine (PS) and phosphatidylglycerol (PG), as well as positively charged, double-chain surfactant carrying a trimethylammonium (TAP) headgroup, directly regulates the surface charge characteristics of liposomes [6]. Electrostatic repulsion between the charged liposomes prevents the prepared liposome dispersion from aggregating and governs the interaction between liposomes and the surrounding biological substances, further influencing the toxicity. Briefly, positively charged liposomes are relatively more toxic than negatively charged ones [18]. Additionally, the size distribution of liposomes plays a less significant role in influencing their toxicity, but largely determines the biodistribution and circulation time of liposomes [7]. Liposomes of sizes ranging from 100 to 200 nm can take advantage of the enhanced permeability and retention (EPR) effect of tumor blood vessels and passively accumulate at the tumor sites (Figure 3), thereby resulting in targeted drug delivery and reduced systemic toxicity [19,20,21].

Efficient loading of therapeutic agents (payload), including hydrophobic, ionic, and zwitterionic ones, into liposomes to obtain a reasonable drug-to-lipid ratio is critical for the drug delivery system [3,23]. The encapsulated molecules by liposomes show improved solubility in aqueous media, particularly for the hydrophobic drugs, enhanced stability and bioavailability, as well as reduced side effects. Efficient drug-loading methods, depending on the nature of drugs, have been developed [24,25,26,27]. Drug-free liposomes can be prepared using hydration–extrusion, sonication, reverse phase evaporation, microfluidics, and supercritical carbon-dioxide-mediated processes [28,29,30]. Drug-loading is performed during liposome formation (passive loading) or afterward (such as active loading). Water-insoluble drugs or therapeutic agents, which are incorporated in the center of the hydrophobic region of the lipid bilayer, are generally encapsulated into liposomes passive way, resulting in high encapsulation effectiveness. During this process, interactions between lipid and drug molecules play a key role, whereas hydrophobic lipid molecules, such as PCs with longer and saturated alkyl chains, favor encapsulation [31]. Hydrophilic drugs can be encapsulated by passive or active loading [25]. For water-soluble weak acids and bases, remote loading of these molecules into liposomes takes advantage of the ion gradient across the bilayer, forming a stable (semi-)solid phase (gel-like) precipitate in the interior of the liposome, thereby resulting in high loading efficiency and avoiding burst/fast release [24,32]. 

The effectiveness of liposomal drug formulation is achieved mainly via the uptake of liposomes by target cells, mediated by liposome adsorption onto the cell surface, followed by cellular uptake via membrane fusion or endocytosis, or other methods [33]. Drugs are released from the liposomal formulation by passive permeation and diffusion, and exert their therapeutic effects [26]. Controlled and prolonged drug release can be achieved by tuning the physicochemical properties of the liposomal carriers, modification with stimulus-responsive and/or targeting moieties, and so on.

Despite the advantages mentioned previously, there are still some problems associated with using conventional liposomes as drug delivery platforms. Their colloidal and in vivo stabilities are vital issues (Figure 2). One problem associated with conventional liposomes, especially the neutral ones, is the colloidal instability of aqueous dispersion over time, giving rise to vesicle fusion and aggregation, causing limited shelf time [34]. After administration, the interactions between liposomes and biomacromolecules or cells in the surrounding biological environment, i.e., plasma proteins and cells, play a key role in the pharmacokinetics of liposomes. Another problem is that liposomes bind to proteins and other biomolecules in the biological fluid (such as serum albumin) via electrostatic, hydrophobic, and other specific interactions, forming a “protein corona” about the surface, which alters the characteristics of the liposomal formulation (size, surface charge, and properties, etc.) [35,36]. The formed complex may disrupt liposomes, promote drug pre-release [37], and determine biodistribution and cellular uptake pathways. The protein corona enables non-specific rapid clearance of administered liposomes from circulation by the mononuclear phagocyte system (MPS) before reaching the target sites other than the MPS tissues (i.e., liver and spleen) [38,39,40], resulting in low efficacy. Characteristics of liposomes, including lipid composition, size, surface charge, fluidity, and hydrophobicity, influence the circulation time [41,42]. 

To overcome the problems associated with using conventional liposomes as drug-delivery carriers, the outer surface of conventional liposomes provides sites for functionalization. Tuning the physiochemical properties, most importantly, the interfacial properties of liposomes by varying lipid composition (the second-generation liposomes), by surface modification with gangliosides or sialic acid (the second-generation liposomes), and surface modification of liposomes with polymers, has been intensively investigated to modify liposome-involved interactions (i.e., liposome–liposome, liposome–serum proteins) [43,44,45,46,47]. Versatile polymers, including natural and synthetic ones, have long been used as drug delivery carriers due to their tailorable structures, properties, and functionalities [48]. Depending on the characteristic of the incorporated polymer, the polymer-modified liposomes show superior performances in improved colloidal stability, prolonged circulation, and targeted and triggered release [49]. Briefly, surface modification of liposomes with polymers uses at least one of the following strategies: polymer grafting on the surface of liposomes and liposomes coated with physically adsorbed polymers (Figure 1b,c). Additionally, multi-functional liposomes have also been engineered by incorporating polymers with more than one specific functionality.

## 3. Polymer-“Grafted” Liposomes

**Figure 4:**
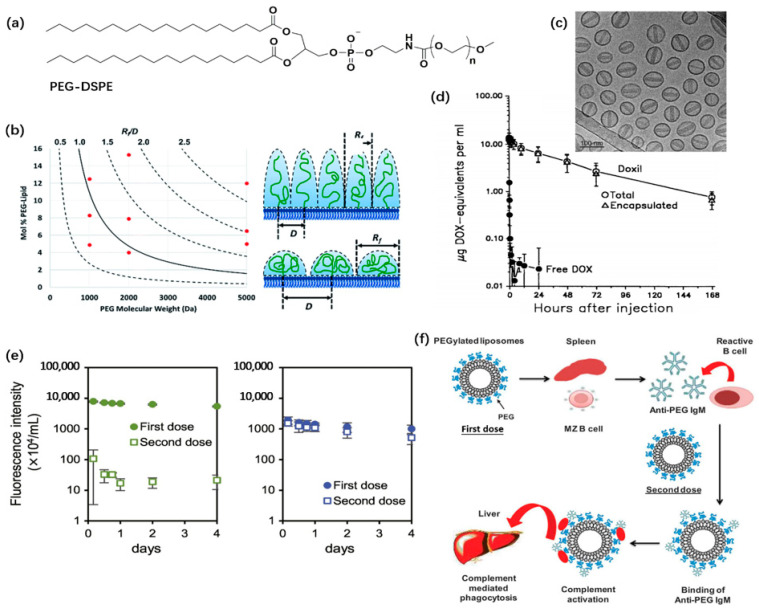
PEGylated liposomal formulation. (**a**) Chemical structure of PEG-DSPE conjugate, where *n* represents the number of repeating units; (**b**) Determination of the configuration of surface-grafted polymer; (**c**) TEM image of PEGylated doxorubicin; (**d**) Pharmacokinetics of PEGylated liposomal doxorubicin (open symbols) and free drug (solid symbols); (**e**) Blood concentration after the first and second doses (solid and open symbols, respectively) for PEGylated liposome induces ABC while poly(sarcosine)-modified liposomes do not cause ABC; (**f**) Mechanism of ABC induced by repeated administration of PEGylated liposomes. The panel (**b**) is adapted with permission from Ref. [54], published by Royal Society of Chemistry, 2018. The panel (**c**) is adapted with permission from Ref. [5]; Copyright © 2012 Elsevier B.V. All rights reserved. The panel (**d**) is adapted from Ref. [62]; Copyright © 1994, American Association for Cancer Research. The panel (**e**) is adapted with permission from Ref. [63]; Copyright © 2020 Elsevier B.V. All rights reserved. The panel (**f**) is adapted from Ref. [64], an Open Access article distributed under the terms of the Creative Commons Attribution License; Copyright © 2019 Mohamed et al.

For polymer-grafted liposomes, hydrophilic polymers bear one nonadsorbing end with the other end attached to the liposome surface either by the “grafting to” approach, where the hydrophobic anchors are inserted into the lipid bilayer mainly in forms of amphiphilic polymer-lipid conjugates (Figure 4a) and bola amphiphiles composed of two hydrophilic chains connected by a hydrophobic domain [50,51], or by the “grafting from” approach through directly grafting polymers from the liposomal surface (liposome-surface-initiated atom-transfer radical polymerization) [52]. The hydrophilic polymers form a thicker hydration layer surrounding the liposome surface, which inhibits the interactions between nearby liposomes, and between liposome and proteins and/or cells in the biological environment by steric, hydration, and/or electrostatic repulsion [34].

The interactions between surface-grafted nanocarriers and biomolecules are determined by the grafting density, molecular weight, and configuration of grafted hydrophilic polymers [53,54]. The configuration of polymers bound to a surface, whether forming a mushroom or polymer brush, is mainly dependent on the ratio between the Flory dimension (*R*_f_) and the averaged distance between adjacent chains (*D*) (Figure 4b) [55].

*R*_f_ = *aN*^3/5^ (1)

*D* = (*A*/*M*)^1/2^ (2)

where *a* is the monomer size or persistence length, *N* is the degree of polymerization, *A* is the area per grafted polymer chain in the bilayer, and *M* is the molar fraction of polymer-lipid conjugates. The *R*_f_/*D* values below and above 1.0 indicate mushroom and brush configurations, respectively [55]. In general, increasing chain length and grafting density is beneficial for suppressing protein adsorption and/or macrophage uptake, reducing liposome uptake by MPS, and obtaining prolonged circulation time. Such liposomes are also referred to as “stealth liposomes”. 

Quite a large number of hydrophilic polymers and their derivatives have been investigated to prolong the circulation of liposomes. PEGylated liposome is the most extensively studied and most successfully commercialized one. The PEGylated liposomal formulations approved include Doxil^®^/Caelyx^®^ for the delivery of doxorubicin (DOX, Figure 4c) and Onivyde^®^ for irinotecan [5]. More are under clinical or preclinical studies.

### 3.1. Stealth Liposomes

Surfaces modified with antifouling polymers can effectively resist the non-specific adsorption of proteins and/or adhesion of cells [56,57], therefore overcoming the problems associated with rapid clearance of conventional liposomes by the RES system. Such polymers include PEG-based materials, zwitterionic polymers, and polyampholytes [56,57]. In general, the antifouling performance of the surface-grafted polymer is positively correlated with the hydration level of the polymeric moieties [57]. The configuration of grafted polymers, whether in brush or mushroom configuration, also greatly influences the physiochemical and biological performance [53,54].

#### 3.1.1. PEGylated Liposomes

PEGylation has been recognized as the “gold standard” for steric protection of liposomes [58]. PEG ((-O-CH_2_-CH_2_-)_n_) is hydrophilic, highly flexible, and inexpensive. The antifouling property of PEG is attributed to its hydrophilic nature and hydration ability under aqueous conditions. In contrast, hydrogen bonding dominates the interaction between water molecules and PEG chains [59], and entropic origin, where the high flexibility of PEG chains will be suppressed upon fouling. The thick hydration layer surrounding the polymers, particularly the tightly bound water molecules lying the outmost interfacial layer, inhibits non-specific protein adsorption. In contrast, the mobility of polymers plays a less significant role in the antifouling property [60]. Consequently, PEGylated liposomes show decreased RES uptake and increased blood circulation time, depending on the molecular weight and content of grafted PEG in the vesicles [61].

The configuration of grafted PEG, as illustrated in Figure 4b, is determined by the molecular weight and grafting density. The PEG extension length is given by Equation (1) for the mushroom configuration and by Equation (3) for the polymer brushes:

*L*_brush_ ≈ *aN*^3/5^/*D*^2/3^ (3)

where *D* is the distance between grafted PEG polymers. For the most widely used conjugation of PEG_2000_ and 1,2-distearoyl-*sn*-glycero-3-phosphoethanolamine (DSPE), the configuration of surface-grafted PEG undergoes a transition from mushroom (3.5 nm) to brush (10 nm, fully PEGylated) when the content of PEG_2000_-DSPE exceeds 4 mol%, and the liposomes transit to micelles when PEG_2000_-DSPE is more than ca. 8 mol% [65].

Incorporating PEG–lipid conjugates may also change the phase state of the lipid bilayers, which in turn changes the permeability, bending modulus of the bilayer, and other properties of the liposomes [65,66,67]. Adding DSPE into distearoylphosphatidylcholine (DSPC) bilayer changes the phase state of the bilayer from gel-phase (<ca. 10 mol%) to tilted gel phase (L*β*′), interdigitated gel phase (L*β*I), untitled gel phase (L*β*), or micellar phase, depending on the content and molecular weight of the PEG moieties [68]. A previous study has demonstrated that the fluid-phase PEGylated liposomes interact more weakly with human serum albumin than those in gel-state [67].

Steric interaction plays an essential role in the improved colloidal stability and favorable pharmacokinetic and therapeutic properties of PEGylated formulation [34,69]. Direct measurement of the normal forces between PEG functionalized DMPC (1,2-dimyristoyl-*sn*-glycero-3-phosphocholine) bilayers (DMPC + 7.5 mol% DMPE-PEG_2000_, either in gel- or in fluid-phase, where DMPE is the abbreviation of 1,2-dimyristoyl-*sn*-glycero-3-phosphoethanolamine) using a surface-force apparatus revealed the presence of roughly exponentially decayed long-range electrostatic (Debye length ca. 20 nm across 0.25 mM NaNO_3_) originating from the phosphate group of PE molecules (Figure 4a) and short-range steric repulsion [70]. Steric interaction between PEG-grafted liposomes increases as PEG-lipid concentration and the molecular weight of the PEG chain increase [55]. Similarly, PEGylated liposomes also exhibit diminished protein and cellular interactions and favorable pharmacokinetic behaviors and therapeutic effects, including very long circulation/elimination time, low RES-uptake, and specific accumulation at tumor sites. The blood clearance curve of liposomes shows a *T*_1/2_ up to 15.3 h for PEG-DSPE incorporated ones and a 4-fold increase compared to those without PEGylation. Meanwhile, the inclusion of PEG-DSPE also decreases the amount of liposome in liver but promotes the accumulation in the tumor tissues [71]. Compared with free doxorubicin drug molecules which are rapidly cleared from blood circulation, the elimination half-life of PEGylated liposomal doxorubicin is ca. 45 h in humans (Figure 4d) [62]. These features account for the broad application of PEGylated liposomes as drug delivery carriers.

Despite these advantages, increasing studies on PEG polymer and PEGylated products also reveal some unfavorable effects. The most significant one is that the administration of PEGylated liposomes can cause complement activation, which leads to an accelerated blood clearance (ABC) phenomenon (Figure 4f) and hypersensitivity reactions upon repeated administration, resulting in decreased therapeutic efficacy [72,73]. Different approaches have been investigated to eliminate complement activation by playing with PEGylated formulations and designing alternative polymers as grafting moieties for long-circulating liposomes.

#### 3.1.2. Accelerated Blood Clearance (ABC)

The first injection of PEGylated liposomes induces the production of PEG-specific Immunoglobulin M (IgM) antibodies by the spleen [74]. Upon the second injection, anti-PEG antibodies can bind to PEGylated liposomes, giving rise to complement activation, which in turn enhances the rapid clearance of vesicles from the systemic circulation by complement-mediated phagocytosis [75]. This phenomenon is called accelerated blood clearance (ABC, Figure 4e,f) [64,76]. The plasma concentrations of PEGylated liposome after the second dose drop dramatically than those after the first dose, as shown in Figure 4e [63]. Moreover, possibly due to the prevalent application of PEG in consumer and pharmaceutical products, anti-PEG antibodies, IgG, and/or IgM have been detected in ~72% of the individuals who have never received PEGylated drug treatment [77,78]. These pre-existing antibodies may also induce allergic reactions after individuals receive the first dose [79].

The ABC phenomenon is influenced by many factors, including the backbone of the polymers, PEGylation of the liposomes, molecular weight of PEG, PEG-lipid linkages, animal species, the time interval between administrations, lipid dose, the physicochemical properties (size and charge, for example) of the formulation, as well as the encapsulated drugs [64,69,73,80,81,82,83]. Studies have shown that, for empty PEGylated liposomes (HSPC/Chol/mPEG_2000_-DSPE = 1.49:1.00:0.134, molar ratio, where HSPC is the abbreviation for hydrogenated soy phosphatidylcholine), no ABC was observed at high doses of >5 μmol PLs/kg, but was observed for low doses of <1 μmol PLs/kg; a time interval of 4–7 days between the first and second doses induces a much more pronounced ABC phenomenon [84]; encapsulation of cytotoxic or tolerogenic agents, such as doxorubicin and rapamycin, also inhibits the ABC phenomenon, possibly due to the released doxorubicin in spleen impairing the production of anti-PEG IgM; prolonging the administration interval from 7 days to 4 weeks also eliminates ABC [73,85,86]. More recently, researchers also found that using high-molecular-weight free PEG to saturate already-existing anti-PEG antibodies offers a window for up to 2 months for the practical application of PEGylated therapeutics [85,87].

More strategies have been developed to address the ABC phenomenon. Inserting ganglioside into PEGylated liposome prevents the immunogenicity of PEG and induces B cell tolerance to subsequent doses, thereby preserving the therapeutic efficacy upon repeated administration [88]. 

As the negative charge on PEG–phospholipid, the most commonly used PEG-incorporating amphiphile has proven to play a key role in complement activation [89], PEG–nonphospholipid conjugates, such as PEG_2000_-conjugated hexadecylcarbamoylmethyl hexadecanoate (HDAS-PEG) and cleavable PEG-cholesterol, and can also lessen or eliminate ABC phenomenon [90,91]. On the contrary, introducing a hydroxyl group at the end of the PEG chain can cause enhanced clearance of the subsequent doses [92].

As alternatives to PEG, a number of hydrophilic polymers have been used to fabricate long-circulating liposomes. Poly(carboxybetaine) (PCB), branched PEG, poly(sarcosine) (PSar, Figure 4e, right panel), polyglycerol, poly(hydroxyethyl-l-asparagine) (PHEA), poly(vinylpyrrolidone) (PVP), poly(*N*,*N*-dimethylacrylamide) (PDMA), poly(*N*-acryloyl morpholine) (PAcM), poly[*N*-(2-hydroxypropyl)) methacrylamide] (HPMA), and poly(2-methyl-2-oxazoline) (PMOX) can effectively reduce or eliminate immunogenic response upon repeated injections [63,80,93,94,95,96]. However, PMOX was proved to induce a pronounced ABC effect in rats [80].

#### 3.1.3. Zwitterionic Polymer-Lipid Conjugates

Compared with uncharged PEG polymers, which form hydrogen bonds with water molecules and cannot completely prevent protein binding [97], zwitterionic polymers interact with water molecules mainly via charge–dipole interaction. Therefore, they are more hydrated than PEG. Water molecules surrounding the zwitterionic moieties are more ordered, forming a physical and energy barrier resistant to the adsorption of biological proteins, such as bovine serum albumin and lysozyme [98]. In contrast to the ABC phenomenon associated with PEGylated liposomes, the highly hydrophilic zwitterionic polymers are expected to exhibit improved immunological safety meanwhile maintaining the stealth properties [99]. Consequently, zwitterionic polymer-grafted liposomes incorporating PCB- and poly-(2-(methacryloyloxy)ethyl phosphorylcholine) (PMPC)-grafted liposomes have been designed and investigated as alternatives to PEGylated ones [34,100].

Cao et al. [100] synthesized superhydrophilic zwitterionic PCB–DSPE conjugate with a sharp polarity contrast between the PCB and alkyl chains of DSPE. The highly hydrophilic PCB brushes can stabilize liposome structure more so than amphiphilic PEG chains. Similar to PEGylated liposomes, PCB_5000/2000_-stabilized liposomes are stable at 4 °C for over half a year, and have a longer blood circulation half-life (ca. 3–7 h) than that of the small unilamellar vesicles (SUVs) of DSPC (<1 h). Moreover, doxorubicin encapsulated in DSPC/PCB_5000_-DSPE 10% eliminated tumors by a single i.v. injection, and cured mice faster than commercial Doxil^®^ [100]. Unlike PEGylated liposomes, the pH-sensitive PCB-stabilized liposomes were internalized into cells via endocytosis with superior cellular uptake and drug release. Meanwhile, surface-grafted PCB avoided the ABC phenomenon and promoted accumulation of drug-loaded liposomes to tumor sites [101].

Taking into consideration of the 2-(methacryloyloxy)ethyl phosphorylcholine (MPC) moieties that resemble the highly hydrated headgroups of PC lipids, which largely account for the extreme hydration lubrication of synovial joints [102,103,104], Lin et al. [34] designed and synthesized PMPC–DSPE conjugates, aiming to improve the colloidal stability of conventional liposomes while simultaneously improving the lubrication behavior of the polymer-grafted surface. Compared with bare HSPC-SUVs, which aggregate shortly after preparation, incorporating 2 mol% DSPE-PMPC can significantly inhibit SUV aggregation for more than 90 days [34]. The PMPCylated liposomes exhibit superior in vivo retention time to the commonly used PEGylated ones. The in vivo intra-articular retention time (half-life, *T*_1/2_) of HSPC/PMPC (2%) is 85 h, much longer than HSPC/PEG (<20 h) and commonly administrated HA (<1 h) [12]; size-dependent retention behavior was also observed for HSPC/PMPC (2%), of which the *T*_1/2_ is 85 h and 18–22 h for diameters of 170 and 80 nm, respectively, but not for HSPC/PEG (17 ± 4 h). More interestingly, PMPCylated liposome-coated surfaces are capable of affording extremely low friction coefficients (at 10^−4^ level) under physiologically high pressures, which is one order of magnitude better than those for PEGylated liposomes (friction coefficients 0.006–0.011), attributed to the higher hydration level of MPC moieties [34]. These results together indicate that PMPCylated liposomes are more appropriate as drug carriers for intraarticular drug delivery and as efficient boundary lubricants for treating osteoarthritis (OA), a degenerative joint disease characterized by slowly disrupted synovial cartilage and undermined lubrication performance [4].

As the hydration level of zwitterionic polymer plays a central role in antifouling ability, recently developed antifouling polymers, such as strong-hydration materials (poly trimethylamine *N*-oxide, PTMAO) [105], amino acid-based zwitterionic polymers prepared from L-serine, lysine, ornithine, asparagine, hydroxyethyl-L-asparagine, glutamine, and repeated glutamic acid and lysine motifs [106,107,108], as well as poly(ectoine) [109], also provide feasible options for polymer-grafted liposome, in the context of developing long-circulating formulations. Moreover, further studies on the immunogenic and toxicological performances of the modified liposomes are highly demanded.

## 4. Polymer-Coated Liposomes

The outer surface of liposomes allows adsorption of polymers through non-specific (electrostatic, dipole-charge, hydrogen-bonding, van der Waal’s) interactions. As a consequence of the adsorbed polymers, the interfacial and physiochemical properties of liposomes are modified, thereby attaining colloidal, biological, and mechanical stability, prolonged circulation half-life, reduced permeability and controlled release of the payload, as well as targeting [45,110,111,112]. Direct adsorption of single-component hydrophilic polymers on the liposome surface, incorporating polymers modified with multiple hydrophobic anchors (“comb-like” structure) into a lipid bilayer, layer-by-layer (LbL) deposition of complementary polymers, and caged liposomes with cross-linked polymers are among the most commonly used approaches.

### 4.1. Liposomes Coated with One Layer of Polymer

**Figure 5:**
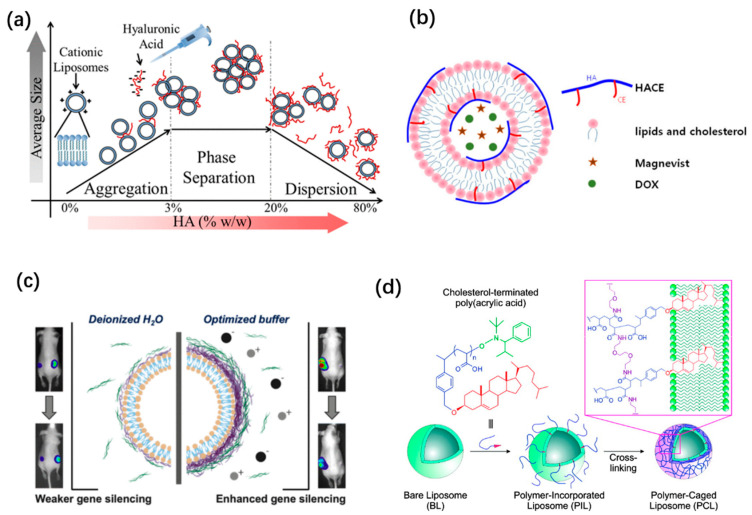
Schematic of liposomes coated with polymers. (**a**) Schematic of the configuration of low-molecular-weight HA/liposome complex. Reprinted with permission from Ref. [113]. Copyright © 2015, American Chemical Society. (**b**) Liposomes modified with hyaluronic acid–ceramide (HA–CE), whereas multiple hydrophobic anchors were introduced to HA. Reprinted with permission from Ref. [114]. Copyright © 2013 Elsevier B.V. All rights reserved. (**c**) Layer-by-layer (LbL) deposition of appositively charged polyelectrolytes. Enhanced therapeutic efficacy was obtained under optimized LbL deposition conditions. Reprinted with permission from Ref. [115]. Copyright © 2019, American Chemical Society. (**d**) Polymer-caged liposomes. Liposomes were modified with poly(acrylic acid)-cholesterol, followed by cross-linking with diamine. Reprinted with permission from Ref. [116]. Copyright © 2007, American Chemical Society.

Coating liposomes with polymer adsorption is performed by adding polymer solution dropwise into already-prepared liposome dispersion, which avoids pre-functionalization of the polymers and is a feasible way of modifying the properties of liposomes. The chemical structure, charge, and molecular weight of polymers predominately determine their interaction with liposomes and the polymer–liposome configuration. As shown in Figure 5a, as the content of low-molecular-weight hyaluronic acid (HA) increases, the configuration of cationic liposome-polymer dispersion changes from aggregation, to phase separation, then dispersion, along with an increase followed by a decrease in the colloidal size (Figure 5a) [113].

Polymers can adsorb on the oppositely-charged liposomes by electrostatic, hydrophobic, and hydrogen-bonding interactions, and bind to zwitterionic PC vesicles, introducing charges to the surface [117]. Therefore, liposomes modified with charged polymers are electrostatically repulsive and are supposed to be colloidal-stable. The coating layer also serves as a barrier controlling the release rate of encapsulated drugs. For example, prolonged release of encapsulated *N*-acetylcysteine was observed for chitosan-coated liposomes compared to the uncoated ones (DPPC/Chol/DPPG, whereas DPPC and DPPG refer to 1,2-dipalmitoyl-*sn*-glycero-3-phosphocholine and 1,2-dipalmitoyl-*sn*-glycero-3-phosphoglycerol sodium salts, respectively) [118].

Strong polymer–liposome interaction may cause leakage of the entrapped agents [119], while weak attachment may cause polymer detachment after injection, leading to a shorter circulation time [120]. For a polymer-coated liposome drug-delivery system, sufficient polymer molecules (saturation concentration) are required to cover the liposomal surfaces. Meanwhile, special attention should be paid to avoid the following problems: inhomogeneous distribution of polymers along with the same vesicle and on vesicles from the same batch [121], dehydration, and fusion of vesicles [122], and bridging or depletion flocculation of the liposome–polymer mixture [123].

Charged polysaccharides, including HA, chitosan, and alginate, have attracted much attention for liposome-based drug delivery systems. In addition to biocompatibility, biodegradability, good aqueous solubility, and charge characteristics, certain polysaccharides also exhibit specific properties without functionalization, such as reinforced mucoadhesion for chitosan and CD44 targeting for HA. Surface-functionalized polysaccharide derivatives could enable the coated liposomes with specific properties. Biocompatible synthetic polymers, such as polyvinyl alcohol (PVA), zwitterionic polymers, and dendrimers [124,125] have also been investigated. It was found that coating with zwitterionic polymer can also inhibit nonspecific protein adsorption to liposomes [124], while polyphenylene dendrimer-coated liposomes (DOPE:Egg PC:Chol = 1:1:1, molar ratio, whereby DOPE is the abbreviation of 1,2-dioleoyl-*sn*-glycero-3-phosphoethanolamine) could inhibit the binding of specific opsonins while increasing the adsorption of proteins that control cellular uptake [125].

To reinforce the attachment between polymer and lipid and form a stable coating layer, one solution is to introduce more anchors to polymer–lipid conjugates [114,120,126]. Liposomes modified with PVA-conjugated hydrophobic anchors (PVA-R) are resistant to aggregation or fusion, and prolong the circulation time in lungs after pulmonary administration, possibly through reduced clearance by macrophage, whereas higher molecular-weight PVA is more effective [120]. To stabilize the coated HA layer, amphiphilic hyaluronic acid derivative and hyaluronic acid–ceramide (HA-CE) were involved in the encapsulation of the anti-cancer drug doxorubicin, and a contrast agent for magnetic resonance imaging, Magnevist, was used. which enabled simultaneous targeted drug delivery and cancer imaging (Figure 5b) [114]. To overcome the problems associated with coating by one polymer layer, the layer-by-layer (LbL) technique (Figure 5c) and polymer-caging (Figure 5d) have also been investigated (Figure 1c).

### 4.2. Layer-by-Layer (LbL)

LbL coating is a simple process achieved by subsequent adsorption materials to form a stable layer on the substrate/surface. Electrostatic attraction is the most commonly used driving force fabricating LbL structures. Other assemblies by virtue of hydrophobic, charge–transfer, host–guest, biologically specific, coordination chemistry, and covalent bonding interactions have also been reported [127]. The versatility of LbL assembly, particularly of the outermost layer, provides a wide range of properties and functions to the drug delivery systems, including but not limited to steric stability, stealth properties, active targeting, and environmental responsiveness [115,128,129].

For the self-assembly of alternatively deposited charged polymers on liposomes, an ion-exchange phenomenon whereby the associated counterions about the substrates are replaced by the next layer of charged polyelectrolytes, the main driving force is entropy gain from the released counterions [130]. The internal charge balance of the system is therefore maintained by the intrinsic lipid/polymer and polymer/polymer ion pairs, and extrinsic polymer/counterion pairs. As surface-charge reversal has been commonly observed in such systems [131,132], charge overcompensation is considered to play a significant role in successfully building the multilayers [133]. However, it was reported that charge overcompensation during the growth of multilayers poly(diallyldimethylammonium chloride)/poly(styrene sulfonate) (PDADMAC/PSS) took place only on the adsorption of polycations, due to the differences lying in the reaction-diffusion ranges of charges from positive and negative polyelectrolytes [134].

Competitive interactions between polyelectrolyte–liposome and oppositely charged polyelectrolytes also drive the formed structures and the colloidal stability of the liposomes. Despite charge reversal revealed by measuring zeta-potential values after each coating, sequential adsorption of biopolyelectrolytes with respectively high and low charge densities, dextran sulfate-sodium salt and poly-L-arginine, onto cationic liposomes composed of 1,2-dioleoyl-*sn*-glycero-3-phosphocholine and 1,2-dioleoyl-3-trimethylammonium-propane (DOPC/DOTAP, 1:1, molar ratio) resulted in the formation of heterogeneous polyelectrolyte patches [135]. The number of polymers adsorbed on the surface is determined by the surface–polymer interaction and is self-regulated due to electrostatic repulsion. Charges on the liposome core control the adsorption amount of polymer in the first layer. However, no template effect was observed for the second and following layers, namely, the amount of adsorbed polymers for the subsequent layers is independent of the liposomal membrane composition [133]. Taking together the “soft” nature of lipid bilayer into consideration, rational selection of a polycation/polyanion pair with reasonable charge densities is of crucial importance to the successful fabrication of multilayers. A summary of representative studies on LbL-coated liposomes for drug delivery is listed in Table 1.

In addition to the properties of polymers, the layer-by-layer assembly is also sensitive to processing parameters. For example, salt concentration and valence in the solution determine the ionization degree of the polyelectrolytes, the Debye length of charges, and the kinetics and thermodynamics of the assembly [115]. Other parameters, such as concentration of polymers in the solution, temperature, titration rate, as well as stirring speed, also play non-negligible roles in the coating process and should be optimized whenever necessary [115,132,138,149]. During the fabricating process, separating the coated particles and free polymers followed by a washing step to remove residual free polymers are essential after the coating of each layer [150].

Compared with liposomes coated with one layer of polymer, the thin multi-layer is more robust with greatly enhanced stability in resisting damages both in the biological environment and from surfactants, lyophilization, spay-drying, etc. [8,151]. The stiffness of the liposomal core, which can be modulated by incorporating cholesterol, directly influences the mechanical properties of the LbL liposomal system, giving rise to a longer elimination lifetime and higher tumor accumulation and penetration obtained with the compliant liposomes (DSPC with 40 mol% Chol) than those of the DSPC-liposomes [152]. Moreover, this multilayer provides more sites for drug encapsulation and regulates the release kinetics of encapsulated drugs to a more significant extent [142,153]. The improved release profile for low-stable drugs was obtained by anionic xanthan and cationic-galactomannan-coated cationic-DODAB liposomes for protein drug delivery. One layer of xanthan and galactomannan slowed down the release of encapsulated epidermal growth factor, which is five times slower than that of plain liposomes [147]. Although the number of layers is essential to developing stabilized formulation and to the pharmacokinetics of encapsulated drugs [140,142], the coverage of polymers in the first layer and the ratio of charged lipids on the vesicle controlled the release rate [119].

### 4.3. Cross-Linked-Polymer-Caged Liposomes

Chemical cross-linking of the polymers coated on the liposome can efficiently prevent dissociating of polymers and make liposomes more stable, but sometimes it is at the expense of controllable releasing of the payload. To address this problem, triggered release is particularly in demand. Lee et al. [116] prepared DPPC-DOPG-Chol-liposomes (51.4:3.6:45, molar ratio, whereby DOPG is the abbreviation of 1,2-dioleoyl-*sn*-glycero-3-phosphoglycerol) modified with cholesterol-poly(acrylic acid) conjugates, followed by cross-linking of PAA with 2,2′-(ethylenedioxy)-bis(ethylamine), forming a remarkably stable “polymer-caged liposome” that preserved the spherical configuration even after free-drying and rehydration, whereas un-crosslinked ones were not stable during long circulation (Figure 5d). More significantly, the residual carboxylate groups allow for the pH-sensitive release of the payload. Later, protease-triggered, polymer-caged liposomes were fabricated using a urokinase plasminogen activator (peptide GSGRSAGC) and poly(acrylic acid) graft copolymer, which was thereafter crosslinked with diamine [154]. The caged liposomes were stable under physiological conditions until interacting with tumor-specific protease and quickly releasing its contents. Polydopamine coating infers a surface with antifouling properties. Awasthi et al. [155] succussed in crosslinking dopamine uniformly on liposomes under physiological conditions, in contrast to the traditional protocol performed under basic conditions, which yield unstable and non-uniform coatings. Implants coated with a stable polydopamine liposome are resistant to biofouling, which enables practical application of polydopamine-coated liposomes.

## 5. Targeting and Stimulus-Responsive Functionalization for Biomedical Applications

Liposomes that can target specific sites or/and are responsive to environmental stimulus are important to mediate liposome–target interaction and drug release, obtaining local, on-demand delivery. Selectively targeted, stimulus-responsive, and dual functionalized liposomes have attracted much attention as drug-delivery vehicles. Polymer-modified liposomal drug-delivery systems for specific disease and tissue targeting are of particular importance for therapeutic applications [156,157].

### 5.1. Targeting

Mucosal drug delivery [158] has enabled targeted delivery to the mucus layer with a prolonged local effect. Chitosan, acquired by partial deacetylation of chitin, is comprised of glucosamine and *N*-acetyl glucosamine linked by *β*-(1-4) glycosidic bonds. The primary amine groups on the chitosan backbone are protonated at a low pH, making chitosan molecules positively charged. More importantly, chitosan can bind to a negative mucus layer and is mucoadhesive, showing enhanced penetration across intestinal and nasal barriers and improved bioavailability of drugs at the diseased sites. For this reason, chitosan-coated liposomes have been examined for administration via intravenous, oral, ocular, and transdermal routes [159,160]. Studies have shown that liposomes coated with chitosan can penetrate through the intestinal mucosa after oral administration, without detaching from the liposome surfaces [161]; when complexed with sodium tripolyphosphate (TPP), chitosan formed a crosslinked coating layer for liposomes encapsulating quercetin, which increased bioavailability and optimized release of quercetin in intestine after oral administration [162].

Targeting tumor-associated compounds, such as CD44 and folic receptor, is crucial to enhance the therapeutic efficacy of anticancer liposomal formulations (Figure 3) [163]. HA, a major component of the extra-cellular matrix (ECM), is biocompatible and non-toxic, therefore, it has a wide application in biomaterials [164]. HA receptor CD44 is a transmembrane protein that maintains a low level in normal cells but is overexpressed in a number of solid tumors [165]. As HA is a targeting agent to cancer cells overexpressing CD44, HA–DPPE conjugates facilitate the recognition of modified liposomes by MiaPaCa2 cells expressing CD44, and the uptake increases with increasing molecular weight of HA from 4800 to 12,000 Da [166]. As HA is negatively charged under biological conditions due to the negative carboxylic residues, HA-coated liposomes show colloidal stability and no significant change in encapsulated drug for >3 months, in contrast to <1 month for the uncoated ones, possibly attributable to the polymer–liposome network [167]. Folate receptor is another tumor marker overexpressed in many cancers, such as ovarian, endometrial, and renal cancers [168]. A commonly used approach to target folate receptors is to conjugate folic acid, a high-affinity ligand, to a polymer via the gamma-carboxyl of folic acid. As a targeting ligand, folic acid (FA) shows unique advantages, such as non-immunogenic, good stability, high specificity, low price, and compatibility with organic solvents. It also has disadvantages, of which the hydrophobicity of folic acid is a big concern. Incorporating folate–PEG–lipid conjugates (FA–PEG–DSPE/DPPE/SA) into liposomes has proven to significantly promote folate receptor-mediated endocytosis, remarkably improve therapeutic effects, and reduce systemic toxicity [169].

The blood–brain barrier (BBB) rigorously blocks drugs from penetrating the brain. By introducing two targeting moieties, including the *D*-peptide ligand targeting nicotine acetylcholine receptors on the BBB and an integrin ligand targeting integrin on the blood–brain tumor barrier and glioma cells, to the modified liposomes encapsulating doxorubicin, a better anti-glioma effect along with prolonged median survival was observed for glioma-bearing nude mice than those treated by liposomes with none or one moiety; deep penetration of the formulation is still limited by high interstitial pressure and dense ECM in tumors [170].

### 5.2. Stimuli-Responsive Polymers

Triggered release of encapsulated therapeutic agents at the desired site is important to achieve high bioavailability and therapeutic efficacy. Specific fusion or destabilization of liposomes as responses to certain pathological changes between normal tissues and the diseased or target sites, or to the changes in environmental conditions between endosome and cytoplasm after uptake by cells through the endocytic pathway, can be utilized to enable effective controlled release and improved therapeutic efficacy of the payload. In combination with the above-mentioned polymer modification techniques, drug-delivery liposomes responsive to one or more stimuli, such as pH, temperature, light, redox, and specific enzymes, have been intensively studied and well-reviewed [171,172,173,174,175].

A reduction in pH has been reported for tumor tissues (pH 6.72–7.01 for human tumor xenograft lines) [176], inflammatory diseases (ca. 0.5 pH unit reduction) [177], or endo/lysosome (pH ~6.2 for early endosome, and 4.6–5.0 for lysosome) [178]. The change in pH can be utilized to modulate the release of encapsulated therapeutic agents from pH-sensitive liposomes by means of destabilization of liposomes or vesicle fusion. A brief summary of representative polymers for liposomal modification is listed in Table 2. Briefly, the strategies for designing polymer include: (1) protonation/deprotonation of charged groups triggered by a change in pH induces hydrophilicity/hydrophobicity transition and sometimes conformational switch of polymers; (2) pH-cleavable PEGylation (dePEGylation) by acid-labile linkers.

The pH-responsive homopolymer-bearing carboxyl groups, such as poly(aspartic), grafted with hydrophobic anchor octylamine (PASP-*g*-C8) [179], has been applied to induce destabilization and/or fusogenicity under weakly acidic conditions. Modification of widely investigated polymers with pH-sensitive moieties for the functionalization of liposomes could combine the functionality of both the polymer (such as long circulation for PEG) and pH sensitivity to the liposomal formulation. Such polymers include pH-sensitive PEG–lipid conjugates (DSPE-PEG-H7K(R2)2/TH/STP and stearoyl-poly(ethylene glycol)-poly(methacryloyl sulfadimethoxine) copolymer (stearoyl-PEG-PSDM) [185,186,187,189], HA derivatives bearing 3-methyl glutarylated (MGlu) units or 2-carboxycyclohexane-1-carboxylated (CHex) units (MGlu-HA or CHex-HA) [181], 3-methylglutarylated hyperbranched poly(glycidol) (MGlu-HPG) [184,198], etc. Liposome-coated MGlu-HA or CHex-HA not only exhibited a high cellular association to high CD44-expressing cells, but also delivered encapsulated drugs into cells via pH-responsive membrane-disruptive ability [181].

For copolymers bearing weakly charged groups and hydrophobic groups, the decreased ionization of the hydrophilic segments induces configuration changing from random coil to collapsed uncharged globular, which in turn changes the surface charge and aggregation of liposomes, as reflected by the zeta-potential and size distribution [190]. Such phenomena that hydrophilic-to-hydrophobic transition of polymers destabilizes liposomal formulation have been reported for poly(styrene-*co*-maleic acid) (PSMA, pK_1_ = 5.27), which undergoes a conformational change from charged random coil to uncharged globular, as a result of the decreased degree of ionization in the carboxylate groups. The pK1 value of PSMA can be adjusted by the molecular weight of the polymer. Consequently, enhanced cytosolic delivery of encapsulated biomolecules through endosome destabilization together with stability in serum, excellent cytocompatibility, and more efficient drug delivery than unmodified liposomes were observed for the PSMA-modified DSPC liposomes [190]. A hydrophobic to hydrophilic transition was reported for the PDMA section of a bola triblock copolymer, PEG_m_-PDPA_n_-PEG_m_. The bola polymer stabilizes liposome at normal pH but destabilizes it and promotes drug release under acidic conditions [51].

**Figure 6:**
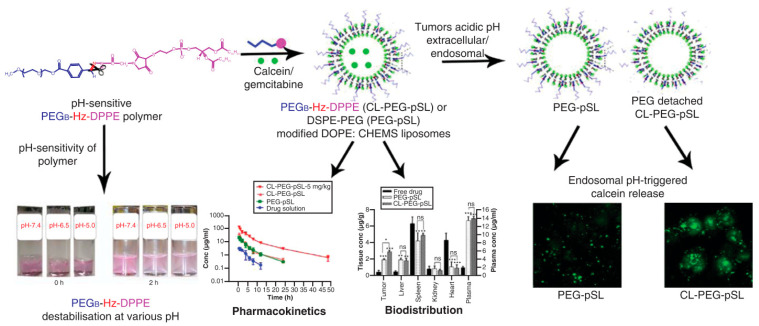
Liposomes modified with pH-sensitive PEG-Hz-DPPE polymers. DePEGylation was achieved by introducing a pH-sheddable hydrazide–hydrazone linker to the PEG–lipid conjugate. Under mild acidic environments, rapid endo/lysosomal escape and enhanced accumulation of drugs in model tumor cells show that cleavable PEGylation is an efficient strategy for cancer therapy. * *p* < 0.05; ** *p* < 0.01; *** *p* < 0.001 versus free drug. Adapted from Ref. [195]. Copyright © 2019, Future Medicine.

Stimulus-responsive dePEGylation is an attractive approach for triggered release (Figure 6). Although the stealth liposomes show a long circulating time and minimal payload leakage during circulation, they may diminish the fusogenic capacity of liposomes and hinder the release of encapsulated drugs at the diseased/target sites (PEG dilemma) [171,199]. To facilitate drug release at the desired location/enhance internalization of the nanocarriers into target cells, cleavage of PEG (dePEGylation) at the desired sites can be made using PEG-(stimuli-responsive linker)-lipid conjugates. Such linkers include not only pH-responsive diortho ester [197], vinyl ether [200,201], hydrazone or hydrazide–hydrazone [14,202], but also reducing agent-responsive disulfide [203] and enzyme-responsive peptides [204]. In addition to promoting extracellular/intercellular release, the dePEGylation approach exposes the liposomal surface, which may cause enhanced liposome–cellular interaction, restored fusogenicity of liposomes, and enablement of endosomal escape, as well as a lowered anti-PEG immune response [171].

Mild hyperthermia has been investigated as an effective method of enhancing local drug release. For thermo-sensitive polymers with a lower critical solution temperature (LCST) near body temperature, the temperature-induced configuration change can be utilized to manipulate drug release from liposomes. Poly(*N*-isopropylacrylamide) (PNIPAAm) is a prototype polymer exhibiting an LCST near 32 °C in an aqueous environment, above which temperature its polymer chain undergoes a coil-to-globule transition [205]. Therefore, PNIPAAm derivatives have been the most popular polymers for thermo-responsive drug delivery. The reported PNIPAAm derivatives and their corresponding LCST include C_12_H_25_–PNIPAAm–COOH conjugate (37 °C) [206], PNIPAAm-DSPE (34 °C in water and 29.8 °C in 0.01 M PBS, pH 7.4) [207], and p(NIPAAm-co-DMAAm)-DSPE (46.9 °C in water and 38.8 °C in PBS) [207]. As PNIPAAm is not biodegradable, poly(*N*-(2-hydroxypropyl)methacrylamide) (PHPMA) polymers have been studied as biodegradable alternatives [208]. Quick thermo-responsive polymer p(NIPAM-*r*-HPMA) (LCST 42 °C), synthesized by a reversible addition-fragmentation chain transfer (RAFT) technique, maintained the stability of liposomes at 37 °C but promoted 70% doxorubicin release within 1 min even at 42 °C with 1% polymer incorporated into the liposome [209]. Other reported thermo-sensitive polymers for liposome modification include a block copolymer of (2-ethoxy)ethoxyethyl vinyl ether (EOEOVE) with anchor group octadecyl vinyl ether (ODVE) (P(EOEOVE-*s*-ODVE)) [210], Pluronic F127 and its derivatives [211], etc.

In addition to pH and temperature, stimuli such as light, redox, magnetic fields, and enzymes have also been reported to trigger drug release from liposomes modified with specific stimulus-responsive molecules [172,212]. Liposomes functionalized with a combination of more than one stimulus or combined stimuli and targeting have also been designed for more effective drug release [213].

## 6. Conclusions and Perspective

In summary, polymer-modified liposomes as drug-delivery carriers have found broad and promising applications by specific polymers. In addition to the clinically approved formulations, much more promising studies have been conducted at lab-scale. Some critical issues should be addressed to bring more liposomal drug-delivery systems into market. More efforts should be put into regulatory matters and scaling-up of complex formulations, sufficient and comprehensive in vitro and in vivo experiments, and a deeper understanding of the mechanism of action for encapsulated drugs.

## Figures and Tables

**Table 1:** Presentative studies on LbL-coated liposomal systems designed for drug delivery.

Liposome Composition	Polycations	Polyanions	Encapsulated Agents	Properties	Ref.
Soya lecithin (24.5 mg), Chol (11.5 mg), stearyl amine (SA, 2 mg)	Chitosan	Polyacrylic acid (PAA)	Paclitaxel (PTX)	Good stability in simulated gastrointestinal fluids after lyophilization, sustained release with a 3-h lag time compared with PTX-liposome, enhanced cytotoxicity by PTX due to cell affinity of chitosan	[136]
DPPC/Chol/DDAB	Chitosan	Alginate	Bovine serum albumin (BSA)	Increased colloidal stability, Enhanced drug encapsulation efficacy, sustained linear release of BSA	[137]
DPPC/Chol/DDAB	Chitosan	Alginate	Recombinant human (rh) osteogenic protein-1/rhOP-1	Locally restricted release of rhOP-1 and resulting effect	[138]
DMPC/DLPA	Chitosan	Dextran sulfate (DXS) or deoxyribonucleic acid (DNA)	1-Hydroxy pyrene-3,6,8-trisulfonic acid (HPTS), alendronate, and glucose	Temperature-dependent release achieved by DNA denaturation	[131]
EYPC/Chol (5:1, mass ratio)	Trimethyl chitosan (TMC)	Pectin	Celastrol	Ideal resistibility to GI conditions, rapid drug release as a response to colonic pH, strong mucoadhesive causing better colon localization and prolonged colonic retention, improved cytotoxicity and visceral toxicity	[139]
EYPC/Chol/EYPG	Poly-L-lysine (PLL)	Poly(L-glutamic acid) PGA	-	Biocompatible and biodegradable polyelectrolytes	[140]
DLPA/DMPC	PLL	Poly-L-aspartic acid (PAsp)	A fluorescent probe, 1-hydroxypyrene-3,6,8-trisulfonic acid (HPTS)	Controlled release of HPTS by the coverage of the first layer and the ratio of DLPA	[119]
DOTAP/DOPE/Chol (35:35:30, molar ratio)	PLL	Hyaluronan or DXS	AZD6244 (selumetinib), an allosteric inhibitor of Mek1/2, and PX-866, a covalent inhibitor of PI3K	Combination chemotherapy, CD44 receptor targeting, hypoxic pH targeting and passive tumor targeting, enhanced efficacy by the synergistic effect	[141]
DPPC/Chol/DDAB (8:4:1, molar ratio)	PLL	Poly(ethylene glycol)-block-poly(L-aspartic acid) (PEG-b-PLD)	DOX and mitoxantrone	Good colloidal stability (>45 days), pH-sensitive, extended systemic circulation time, diminished burst release	[142]
DSPC/POPG/Chol (56:5:39, weight ratio)	Poly-L-arginine (PLA)	DXS	DOX	High drug-load retention, stable stored under room temperature after lyophilization	[143]
DSPC/Chol/POPG (56:39:5)	PLA	siRNA	DOX-loaded liposome, siRNA-loaded film	Extended serum half-life of 28 h, enhanced efficacy by a synergistic effect between SiRNA and doxorubicin	[144]
Soya lecithin (24.5 mg), Chol (11.5 mg), SA (2 mg)	PAH	PAA	PTX	Stable lyophilized formulation in simulated GI fluids, sustained release for 24 h, 4.07-fold increase in oral bioavailability compared to free drug, comparable antitumor efficacy with improved safety as opposed to i.v. Taxol^®^, target potential	[145]
Egg PC/Chol/SA (7:3:1, molar ratio)	PAH	PAA	Amoxicillin and metronidazole	Prolonged drug release in simulated gastric fluid, improved efficacy	[146]
DODAB	Galactomannan (GMC, a neutral polymer)	Xanthan (XAN)	Epidermal growth factor (EGF)	Up to 5 times the sustained release of EGF at a first-order rate of 0.005 min^−1^	[147]
Chol/DSPC/POPG (2:6:2, mass ratio)	poly(*β*-amino ester)	HA	DOX	pH-triggered drug release, CD44 targeting, improved therapeutic effect and reduced side effects	[148]

Chol: cholesterol; DMPC: 1,2-dimyristoyl-*sn*-glycero-3-phosphocholine; DLPA: dilauroyl phosphatidic acid; DPPC: 1, 2-dipalmitoyl-*sn*-glycero-3-phosphocholine; DDAB: dimethyldioctadecyl-ammonium bromide; EYPC: egg yolk L-α-phosphatidylcholine; EYPG: egg yolk L-*α*-phosphatidyl-DL-glycerol; DOTAP: dioleoyl-3-trimethylammonium propane; DOPE: 1,2-Dioleoyl-*sn*-glycero-3-phosphoethanolamine; DSPC: 1,2-distearoyl-sn-glycero-3-phosphocholine; POPG: 1-palmitoyl-2-oleoyl-*sn*-glycero-3-phospho-(1′-rac-glycerol) sodium salt; DOPC: 1,2-dioleoyl-*sn*-glycero-3-phosphocholine; DODAB: dioctadecyl ammonium bromide; SA: stearyl amine.

**Table 2:** pH-responsive polymers for the modification of liposomes for drug delivery.

Polymer	Responsive pH	Composition of Vesicles	Responsive Groups	Payload	TARGETING SITE	Properties	Ref.
**Polymers/Modified Polymers**							
Octylamine-*graft*-poly (aspartic) (PASP-*g*-C8)	5.0	Lecithin/Chol/PASP-*g*-C8 (48:12:x, mass ratio)	Carboxyl groups	Cytarabine (CYT)	Tumor cells	pH-induced destabilization of the liposomes, good biological stability, strong toxicity to tumor cells, and low effect on normal ones	[179]
Lipid-poly(2-ethylacrylic acid) (PEAA-C10)	4.5	PC/Chol/PEAA	Carboxyl group	Calcein	Potentially for tumor or localized infection	pH- and temperature-sensitive due to lipid-anchored PEAA and due to the introduction of diisopropylamide	[180]
MGlu-HA-C10, CHex-HA-C10	pKa = 5.37–6.70	EYPC/HA derivatives	Carboxyl groups	Doxorubicin (DOX)	Interior of cells	High molecular affinity to highly CD44-expressing cells and delivering drugs to the interior of cells as a result of pH-responsive membrane disruptive ability in endo/lysosomes	[181]
MGlu-HA and Chex-HA with anchor moieties (MGlu-HA-A14 and CHex-HA-A14)	-	EYPC/HA derivatives (7/3, *w*/*w*)	Carboxyl groups	A model antigenic protein ovalbumin (OVA)	Antigen-presenting cells (APCs), cytoplasm	Cytoplasmic delivery of OVA into dendritic cells, promoted Th1 cytokine production from these cells with CHex-HA-A	[182]
Succinylated poly(glycidol) (SucPG)	-	EYPC/SucPG (9:1, 8:2, and 7:3, mass ratio)	Carboxyl groups	Calcein	Cytoplasm	Transferring the content into cytoplasm by fusing with membranes of endosome and/or lysosome potentially with high stability and high efficiency	[183]
3-methyl-glutarylated hyperbranched poly(glycidol)s (MGlu-HPGs-C10)	6.5	EYPC/polymer = 7/3, *w*/*w*	Carboxyl groups	Pyranine	Cytosol of DC2.4 cells	pH-sensitivity-inducing content release at mildly acidic pH, efficient drug-delivery to cytosol of DC2.4 cells	[184]
DSPE-PEG-H_7_K (R_2_)_2_	6.8	DOPE/CHEMS/DSPE-PEG or DSPE-PEG-H_7_K (R_2_)_2_	H_7_ sequence	DOX	Glioma	Tumor-specific pH-triggered DOX release under acidic conditions	[185]
DSPE-PEG_2000_-TH	ca. 6.5 (pKa of the imidazole ring)	Chol/SPC/DSPE-PEG_2000_ or DSPE-PEG_2000_-TH (33:59:2:6, molar ratio)	Imidazole ring in histidine	Paclitaxel (PTX)	Endoplasmic reticulum and Golgi apparatus, tumor cell	86.3% tumor inhibition rate in mice	[186]
DSPE-PEG_2000_-STP	5.8	SPC/Chol/STP-PEG2000-DSPE/DOX (8:2:2:1, mass ratio)	-Lys-Asp-Glu-Glu-segment	DOX	Cytoplasm	Enhanced recognition ability and peneration by formation of α-helix at Lys-Asp-Glu-Glu-segment in the *N*-terminal in the presence of protons	[187]
R6H4-C18	6.4	SPC/Chol (20:1, mass ratio), 2.5 mol% R6H4-C18	Arginine and histidine	PTX	Tumor	Enhanced cellular uptake and intracellular drug delivery	[188]
**pH-responsive block copolymer**							
Stearoyl-PEG- poly(methacryloyl sulfadimethoxine) copolymer (stearoyl-PEG-PSDM)	7.0	mPEG-DSPE/stearoyl-PEG-PSDM/lipid	Sulfadimethoxine		Cancer	Stable in serum, undergoing rearrangement in tumor-like environment, high intracellular drug delivery	[189]
Poly(styrene-*co*-maleic acid) (PSMA)	pK_1_: 5.27	DSPC/SMA (20:1, molar ratio)	Carboxylate groups pK1: 5.27		Cytoplasmic delivery	Configurational transition of the polymer below pK1 leading to disruptive lipid bilayer of erythrocytes, enhanced cytosolic delivery of encapsulated biomolecules through endosome destabilization together with stability in serum, excellent cytocompatibility, and efficient drug delivery than unmodified liposomes	[190]
Methoxy-poly(ethylene glycol)-*b*-poly(N-2-hydroxypropyl methacrylamide-co-histidine)-cholesterol [mPEG-P(HPMA-*g*-His)-Chol)]	5.0	mPEG-P(HPMA-*g*-His)-chol /DPPC (1:34, molar ratio)	Imidazole ring of histidine	DOX	Extracellular matrix (ECM) of tumor	ECM targeting, rapid drug release in an acidic environment, preferential tumor accumulation	[191]
PEG_m_-PDPA_n_-PEG_m_ triblock copolymers	ca. 6.2	HSPC or DOPC/Chol/PEG_m_-PDPA_n_-PEG_m_ in various molar ratios	Tertiary amine groups	DOX	Tumor	pH-controllable drug release due to escape of the bola polymer from liposome at acidic pH as a result of hydrophobic to hydrophilic transition of the PDPA segment	[51]
C_7_H_15_-AZO-*b*-PDPA_n_-*b*-mPEG	6.0	HSPC/DOPC/Chol/C_7_H_15_-AZO-*b*-PDPA_n_-*b*-mPEG, with different ratios	Tertiary amine groups	DOX	-	pH- and photo-dual responsive	[192]
**dePEGylation**							
mPEG-Hz-CHEMS	5.5 [193]	Soy PC/Chol/ mPEG2000-Hz-CHEMS/PTX (90:10:3:3)	Hydrazone	-	-	Prolonged circulation time and almost eliminated ABC phenomenon	[194]
PEG_B_-Hz-DPPE	5.0	DOPE/DSPC/CHEMS/Chol/PEG_B_-Hz-DPPE (4:2:2:2:0.5, molar ratio)	Acid labile hydrazide–hydrazone hybrid bond	Calcein/gemcitabine	Tumor	Simultaneous long circulation and pH sensitivity, increased tumor aggregation	[195]
mPEG_2000_-Hz-stearate (PHS)	<6.5	SPC/cholesterol/PHS	Hydrazone bond	-	Tumor	Stronger pH sensitivity than that of PEG_2000_-Hz-PE, superior cellular uptake and endosomal escape	[196]
mPEG_2000_-Hz-Chol	5.5	S100PC/Chol/mPEG2000-Hz-Chol (90:10:3)	Hydrazone	PTX	Breast cancer cells	Highly sensitive to mild acidic environment, accumulative drug release and enhanced cellular uptake at pH 5.5	[193]
PEG-diortho ester-distearoyl glycerol (POD)	5.5	POD/DOPE (1:9)	Diortho ester	ANTS and DPX	-	Stabilized liposome in serum and blood circulation, sensitive to acidic environment but stable in neutral pH	[197]

MGlu-HA: 3-methylglutarylated hyaluronic acid; Chex-HA: 2-carboxycyclohexane-1-carboxylated hyaluronic acid; TH: an engineered a-helical cell penetrating peptide AGYLLGHINLHHLAHL(Aib)HHIL-NH_2_; PDPA: poly(2-(diisopropylamino) ethylmethacrylate; STP: a peptide SKDEEWHKNNFPLSP; ANTS: 8-aminonaphthalene-1,2,3-trisulfonic acid; DPX: *p*-xylenebis(pyridinium) bromide.

## Data Availability

Not applicable.
